# Glucocorticoids Induce a TH2 Response *In Vitro*

**DOI:** 10.1155/1998/73401

**Published:** 1998

**Authors:** Francisco Ramírez

**Affiliations:** Medical Research Council, Cellular Immunology Unit Sir William Dunn School of Pathology University of Oxford South Parks Road Oxford OX1 3RE UK

**Keywords:** Activation, CD4^+ T cells, cytokines, glucocorticoids

## Abstract

Purified rat CD4^+^ T cells were activated *in vitro*, by the polyclonal mitogen Concanavalin A (Con A) or by mixed lymphocyte reaction (MLR), in the presence or absence of the glucocorticoid dexamethasone (DEX). They were then expanded in IL-2 and subsequently restimulated, this time in the absence of the hormone. The results indicate that the exposure of the cells to DEX in the primary stimulation changed the cytokine synthesis induced by the secondary stimulation. IL-4 production was increased by the pretreatment whereas synthesis of IFN-*γ* was diminished. Addition of DEX in the second activation suppressed all cytokine production. In brief, the transient presence of glucocorticoids in the culture induces a change in the pattern of cytokine production but the continuous presence causes inhibition of cytokine synthesis. Further studies in which IL-4 was used together with DEX showed that the cytokine potentiated the effect of the hormone.

The data here presented suggest that glucocorticoids and the neuroendocrine system may be expected to have long-term immunological effects as well as short-lived immunosuppressive ones. High concentration of glucocorticoids suppress cytokine production but when steroids return to basal levels the immune response is directed in a way that favors Th2-type reactions. Possible implications regarding the immune response to pathogens and autoantigens are discussed.


*Developmental Immunology*, 1998, Vol. 6, pp. 233–243
Reprints available directly from the publisher
Photocopying permitted by license only

© 1998 OPA (Overseas Publishers Association)
N.V. Published by license under the
Harwood Academic Publishers imprint,
part of the Gordon and Breach Publishing Group
Printed in Malaysia

### Glucocorticoids Induce a Th2 Response *In Vitro*

FRANCISCO RAMÍREZ

*Medical Research Council, Cellular Immunology Unit, Sir William Dunn School of Pathology, University of Oxford, South Parks Road, Oxford OX1 3RE.*

*(Received 15 August 1996; In final form 23 August 1997)*

Purified rat CD4+ T cells were activated *in vitro*, by the polyclonal mitogen Concanavalin A (Con A) or by mixed lymphocyte reaction (MLR), in the presence or absence of the glucocorticoid dexamethasone (DEX). They were then expanded in IL-2 and subsequently restimulated, this time in the absence of the hormone. The results indicate that the exposure of the cells to DEX in the primary stimulation changed the cytokine synthesis induced by the secondary stimulation. IL-4 production was increased by the pretreatment whereas synthesis of IFN-$\gamma$ was diminished. Addition of DEX in the second activation suppressed all cytokine production. In brief, the transient presence of glucocorticoids in the culture induces a change in the pattern of cytokine production but the continuous presence causes inhibition of cytokine synthesis. Further studies in which IL-4 was used together with DEX showed that the cytokine potentiated the effect of the hormone.

The data here presented suggest that glucocorticoids and the neuroendocrine system may be expected to have long-term immunological effects as well as short-lived immunosuppressive ones. High concentration of glucocorticoids suppress cytokine production but when steroids return to basal levels the immune response is directed in a way that favors Th2-type reactions. Possible implications regarding the immune response to pathogens and autoantigens are discussed.

*Keywords:* Activation, CD4+ T cells, cytokines, glucocorticoids

### INTRODUCTION

Understanding the influence of glucocorticoids on the immune system is important for two reasons: first, corticosteroids are used as immunosuppressive and anti-inflammatory agents in organ transplantation and in the treatment of many autoimmune and inflammatory diseases. Second, it is not commonly recognised that, in addition to their pharmacological actions, these steroids are natural hormones that play an essential physiological role in the regulation of the immune system. Release of endogenous glucocorticoids protects us against our own defense mechanisms by preventing an excessive immunological reaction (Munck et al., 1984). During the course of an immune response corticoid secretion from the adrenal glands is increased and this mechanism limits the magnitude of the inflammatory reaction to an immunogenic stimulus (Besedovsky et al., 1975). Following activation of the immune system several soluble factors that activate the neuroendocrine system are released (Besedovsky et al., 1981). Partial failures in this interaction between the immune and neuroendocrine systems contribute to the pathogenesis of certain autoimmune and inflammatory experimental diseases like thyroiditis, arthritis and experimental allergic encephalomyelitis (EAE) (Kroemer et al., 1988; MacPhee et al., 1989; Sternberg et al., 1989; Mason et al., 1990). There are also evidences that patients with rheumatoid arthritis show a defective regulation of corticosteroids production (Chikanza et al., 1992).

Our interest in the role of corticosteroids in the regulation of the immune response originated from the study of EAE in Lewis rats. EAE is an animal model of autoimmune disease, similar to multiple sclerosis, that can be induced in the appropriate animal strains after immunization with myelin basic protein (MBP) in complete Freund's adjuvant. EAE in Lewis rats is a monophasic disease characterized by a single episode of paralysis, caused by the action of $CD4^+$ T lymphocytes on the nervous system, followed by spontaneous recovery. Animals that have recovered are refractory to attempts to induce further episodes of disease (Zamvil and Steinman, 1990). The mechanisms responsible for the spontaneous recovery and the maintenance of the refractory state, although object of intense research, are not completely understood. One mechanism that controls the course of EAE is neuroendocrine-mediated immunoregulation. It has been shown that endogenously produced corticosterone plays an essential role in the recovery of rats from EAE: Unlike normal rats, adrenalectomized animals do not recover spontaneously but instead develop a progressive paralysis with a fatal consequence.

If these animals are administered corticosterone, they recover and become refractory to the development of new episodes of disease (Levine et al., 1980; MacPhee et al., 1989). The spontaneous recovery of rats from EAE probably occurs because corticosteroids, which are found at high levels in the sera of paralyzed animals, acutely depress the autoimmune response through their immunosuppressive effects. However, animals that have recovered from disease have normal serum steroid levels indicating that corticosteroids are not directly responsible for the maintenance of the refractory phase of the disease. These findings suggest that, in addition to their acute immunosuppressive actions, corticosteroids may display a more subtle and long-lasting effect on the immune response. As EAE elicited by MBP in the Lewis rat is a cell-mediated disease involving T cells that produce IL-2 and IFN-$\gamma$ (Sedgwick et al., 1989), the refractory phase of EAE could in principle be explained if it could be shown that corticosteroids inhibit proinflammatory cytokine production and induce the production of cytokines that inhibit cell-mediated immune responses (Mason, 1991).

Corticosteroids inhibit the production of many interleukins during T-cell activation (Gillis et al., 1979; Snyder and Unanue, 1982; Arya et al., 1984; Beutler et al., 1986; Waage et al., 1990; Wu et al., 1991). The question we want to answer in the experiments here described is whether in situations where corticosteroid concentration is increased, therapeutically or physiologically, there are any long-term effects produced by the hormone. To elucidate the answer, we have used a similar approach to that employed by others to study the effects of the presence of some cytokines on the development of the immune response (Swain et al., 1988). Cells were activated in the presence of the glucocorticoid analogue dexamethasone (DEX) and cytokine production was examined after a second round of *in vitro* stimulation in the absence of the hormone. This experimental design mimics the *in vivo* situation in EAE, and also during an immune response to a foreign antigen, where the increases in blood corticosteroid levels are transient. We have found that the primary stimulation of $CD4^+$ T cells in the presence of DEX induces on a secondary activation the production of more IL-4 protein and the synthesis of less IFN-$\gamma$. We observe this effect on cells activated by Con A and in the MLR.

### RESULTS

### The Presence of Dexamethasone During Primary Con A Activation Increases IL-4 mRNA in $CD4^+$ T Cells After Secondary Stimulation in the Absence of the Hormone

Although glucocorticoids inhibited IL-4 production in a primary activation (Wu et al., 1991; Byron et al., 1992; Ramírez et al., 1996), a different result was obtained when DEX-treated cells were activated a second time in the absence of the hormone (Ramírez et al., 1996). This protocol, shown in Figure 1, was designed to examine whether corticosteroid primes the cells to produce a particular cytokine response on restimulation and also to mimic to a degree the *in vivo* situation in EAE, where only a transient increase in blood corticosterone levels occurs.

Measurements were made of the levels of IL-4 mRNA from cells cultured in the presence or absence of DEX by RT-PCR analysis. Figure 2 shows that $CD4^+$ T cells cultured with the steroid during the primary activation and expansion periods (lanes 7-9) expressed approximately tenfold higher levels of IL-4 mRNA after secondary activation than cells cultured in the absence of DEX (lanes 1-3). In a previous paper, it was shown that mRNA for IL-10 and IL-13 showed the same temporal variation as that for IL-4 (Ramírez et al., 1996). Similar results to the one shown in Figure 2 are obtained when cells are in the presence of the glucocorticoid only during the expansion phase (data not shown): It is evident that to induce an increase in IL-4 mRNA, it is not necessary to include the hormone in the culture from the primary activation to the expansion phase.

To exclude the possibility that the mechanism of IL-4 mRNA induction shown in Figure 2 is due to carry over of the hormone from the expansion phase into the second activation, DEX was added intentionally in the secondary activation. In this condition, a strong reduction in the amount of IL-4 mRNA was observed in DEX-treated cells (lanes 10-12) and untreated cultures (lanes 4-6). IFN-$\gamma$ production was also strongly inhibited when DEX was added in the

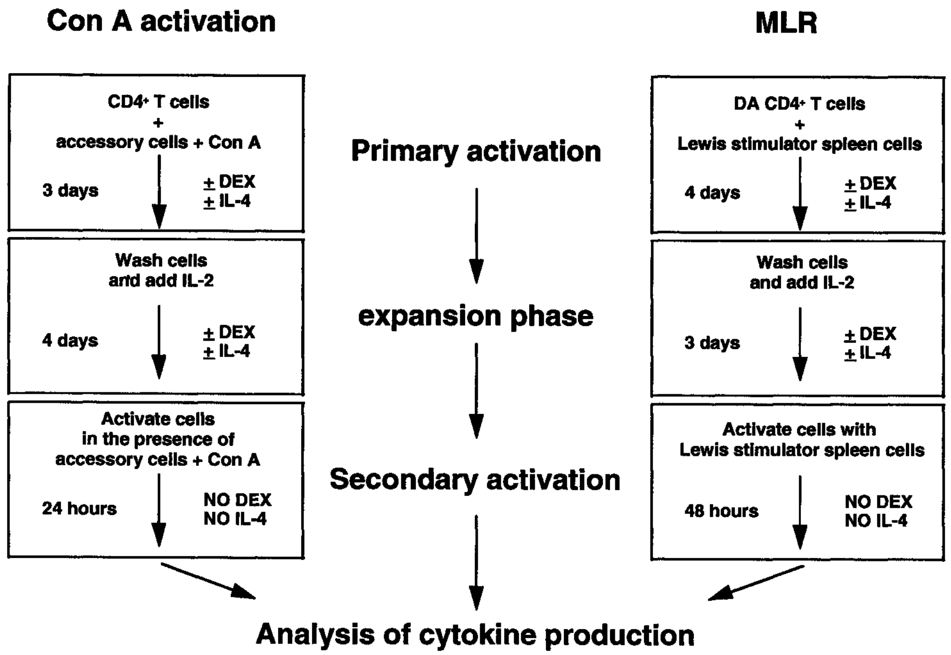

FIGURE 1 Experimental design to analyze long-lasting effects of dexamethasone on cytokine production. T-cell activation was performed by Con A or MLR. To analyze DEX effects on Con A-activated cells the following protocol was adopted: $CD4^+$ T cells ($10^6$/ml) were cultured with accessory cells ($10^6$/ml) and incubated with 5 $\mu$g/ml Con A in the presence of different combinations of DEX ($10^{-8}$ M) and rIL-4 (500 U/ml) for 3 days (primary activation phase). The cells were washed and cultured ($10^5$/ml) for 4 days in the presence of rIL-2 (50 U/ml), some cultures were supplemented with different combinations of DEX ($10^{-8}$ M) and rIL-4 (500 U/ml) (expansion phase). Cells were recovered and restimulated with 5 $\mu$g/ml of Con A in the presence of accessory cells and in the absence of DEX and rIL-4 (secondary activation phase). After 24 hr of the secondary activation, RNA was extracted from the different cell cultures and supernatants were removed for cytokine analysis. To analyze DEX effects on MLR, the protocol was identical to the one described before but cell densities were 2.5 $\times$ $10^6$ responder cells/ml and 2.5 $\times$ $10^6$ stimulator cells/ml on the primary activation. During the expansion phase, cells were cultured at $10^6$ cells/ml. In the secondary activation, stimulator cells densities were 2.5 $\times$ $10^6$ cells/ml, but responder cell densities was reduced to $10^6$ cells/ml. Supernatants were removed at 48 hr of culture.

secondary activation (data not shown), indicating that the presence of glucocorticoids in a primary or secondary activation inhibits the production of IL-4 and IFN-$\gamma$.

### The Effects of Dexamethasone on IL-4 mRNA Levels are Reproduced at the Level of Cytokine Secretion

In the previous experiment (Figure 2), IL-4 production was analyzed at the level of RNA due to the lack of an assay for rat IL-4 protein. An anti-rat IL-4 mAb, which blocks the biological activity of rat IL-4, was obtained recently in our laboratory. It is now possible to quantitate the IL-4 protein in different supernatants testing their capacity to induce the upregulation of MHC class II expression on purified B cells. To assess the specificity of the bioassay, the anti-IL-4 mAb OX-81 is added to the cultures (Ramírez et al., 1996). The levels of IL-4 protein in the supernatants from DEX-treated CD4+ T-cell cultures were assayed and this is shown in Figure 3. Cells treated with DEX during the primary activation and expansion stages produce more IL-4 after secondary activation than control cultures not treated with DEX (undetectable levels). In this experiment, IL-4-treated cells were included as a positive control for the assay, because it is known that IL-4 upregulates its own production (Swain et al., 1990). IL-4- and DEX-treated cells were also included to analyze the effect of the addition of both reagents together in the synthesis of IL-4. As expected, cells activated and expanded in the presence of rIL-4 alone or rIL-4 and DEX together produced higher amounts of IL-4 than the control cultures or DEX-treated cells. Although not shown, cells activated and expanded in the presence of rIL-4 or rIL-4

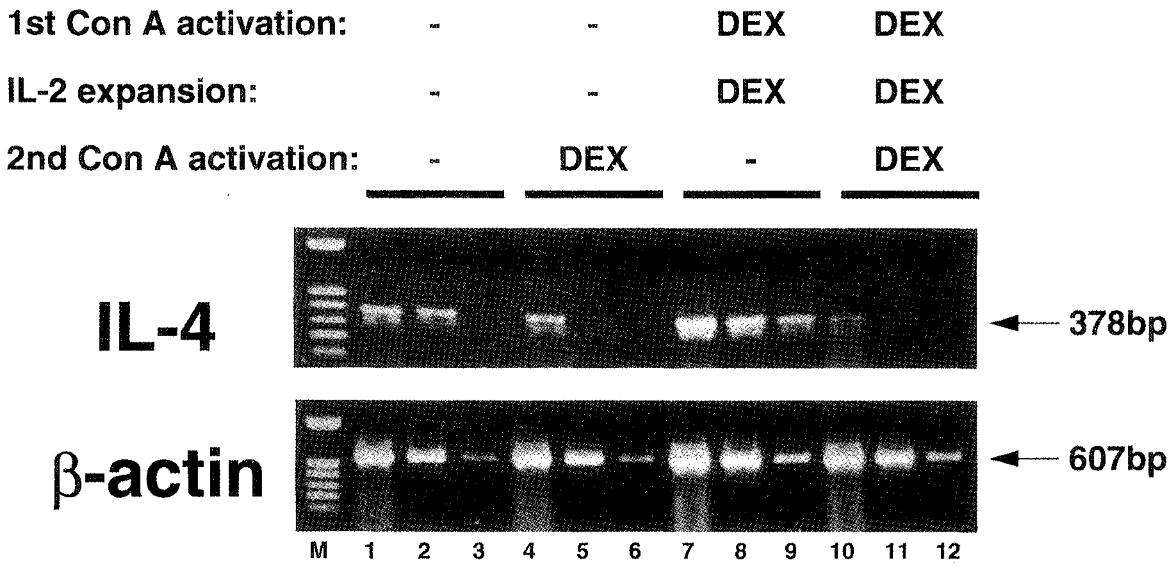

FIGURE 2 Dexamethasone increases IL-4 mRNA levels in Con A-activated CD4+ T cells after secondary activation. Purified rat CD4+ T cells from LN were activated ($10^6$/ml) with Con A (5 $\mu$g/ml) in the presence of accessory cells. After a 3-day activation period, the cells were maintained in rIL-2 for 4 days. Cells were cultured with different combinations of DEX and rIL-4 during the primary activation and expansion periods as described in Materials and Methods (see Fig. 1). Cells were reactivated with Con A in the presence of accessory cells. After a 24-hr activation period, total cellular RNA was extracted and analysis of IL-4 and $\beta$-actin mRNA levels were performed by the RT-PCR technique as described in Materials and Methods. Three tenfold dilutions from each cDNA sample were amplified by PCR (neat, 1/10, and 1/100). In the gel electrophoresis, lanes 1-3 represent amplified products from increasing dilutions of cDNA from control treatment cells, lanes 4-6 amplified products from cDNA from control treatment cells treated during the secondary activation with $10^{-8}$ M DEX, lanes 7-9 amplified products from cDNA from cells cultured during the primary activation and expansion phases in the presence of $10^{-8}$ M DEX, and lanes 10-12 amplified products from cDNA from cells cultured during the primary activation, expansion phases and secondary activation in the presence of $10^{-8}$M DEX. (M: molecular weight markers).

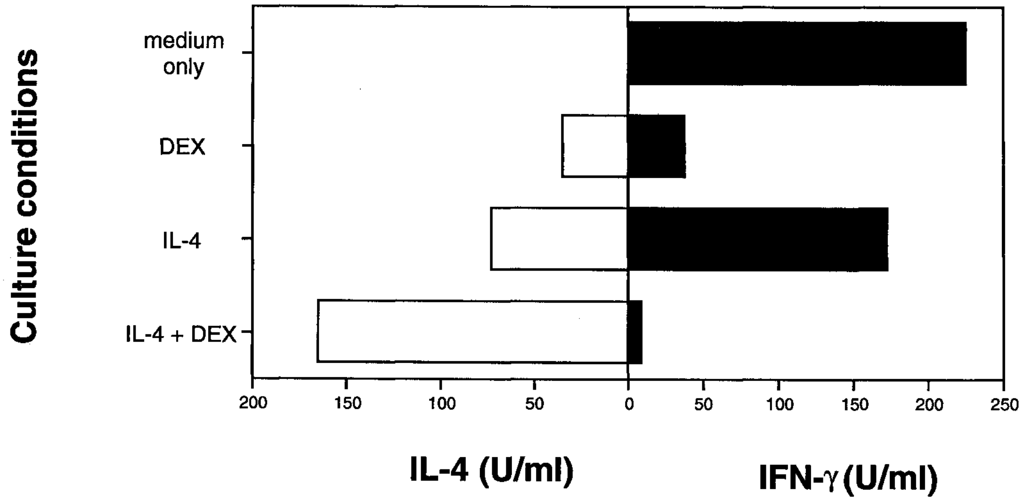

FIGURE 3 Dexamethasone increases IL-4 protein synthesis in Con A-restimulated CD4+ lymphocytes. Purified rat CD4+ lymphocytes from LNC and thoracic duct were activated as described for Fig. 2 and after 24 hr of the secondary activation supernatants were removed for analysis of IL-4 protein by the class II induction assay on B cells, as described in Materials and Methods. The results are depicted as units of IL-4/ml of supernatant calculated by comparison with a rIL-4 standard curve.

and DEX together showed higher IL-4 mRNA levels than DEX-treated cells.

### CD4+ T Cells Activated by Con A in the Presence of Dexamethasone in the Primary Activation Yield Less Thl Cytokines on Secondary Stimulation Than Do Controls

Following the observation that prior activation of CD4+ T cells in the presence of DEX lead to an increase in Th2 cytokine synthesis after secondary activation (Figure 2; Ramírez et al., 1996), an analysis was made of the production of Thl cytokines. Experiments were also carried out to examine the effects on Thl cytokine production of adding rIL-4 in the primary activation and expansion phases either alone or in conjunction with DEX. The results are shown in Figure 3 (only IFN-γ production is depicted from a particular experiment) and Figure 4 (from several experiments including IFN-γ, TNF and IL-2 analysis). Cells cultured in the presence of DEX during the primary activation and expansion stages showed reduced IFN-γ production on subsequent reactivation in the absence of the hormone (31% of control levels). When rIL-4 alone was included in the cultures, CD4+ T cells synthesized similar levels of IFN-γ after secondary stimulation to cells activated by Con A alone. However, if the cells were cultured in the presence of rIL-4 and DEX, there was a striking inhibition of the production of this cytokine (85% inhibition) after secondary stimulation. The lack of inhibition of IFN-γ production by rIL-4 observed in these experiments is in contrast to data published by others (Swain et al., 1990). This point will be discussed further later in this paper.

For TNF production, it was observed that the presence of DEX in both the primary activation and IL-2 expansion phases decreased the production of this cytokine (66% inhibition) (Figure 4). In contrast to the effects observed on IFN-γ production, the addition of rIL-4 to the cultures during the primary activation and expansion phases showed an inhibitory effect on TNF synthesis (71% inhibition), and this effect was stronger when the rIL-4 was supplemented with DEX (86% inhibition).

IL-2 levels were also determined for the same cultures as those assayed for IFN-γ and TNF (Figure 4). DEX treatment did not produce a marked inhibitory effect on IL-2 synthesis in the second stimulation (24% inhibition). The presence of rIL-4 during the primary activation resulted in inhibition of IL-2 production on secondary stimulation (63% inhibition)

and this effect was enhanced in the presence of DEX (98% inhibition).

### The Presence of Dexamethasone During Primary T-Cell Activation in a MLR Increases IL-4 Production in CD4+ T Cells After Secondary Stimulation in the Absence of the Hormone

In the previous experiments, cytokine production was analyzed after Con A activation. It was important to show that the effect of glucocorticoid on cytokine production is independent of the mechanism of T-cell activation. To address this point, analyses of cytokine production were made after MLR activation following the same protocol employed for Con A activation (Figure 1). Figure 5 shows that DEX-treated cells produce more IL-4 than untreated cells (no IL-4 was detected), but IL-4 is a better inducer of IL-4 than DEX. DEX and IL-4 added separately or together during the primary MLR reduce strikingly IFN-γ production by CD4+ T cells on secondary MLR activation (Figure 5). The result obtained treating MLR-activated cells with IL-4 is different to the one obtained by Con A activation, where no inhibition in

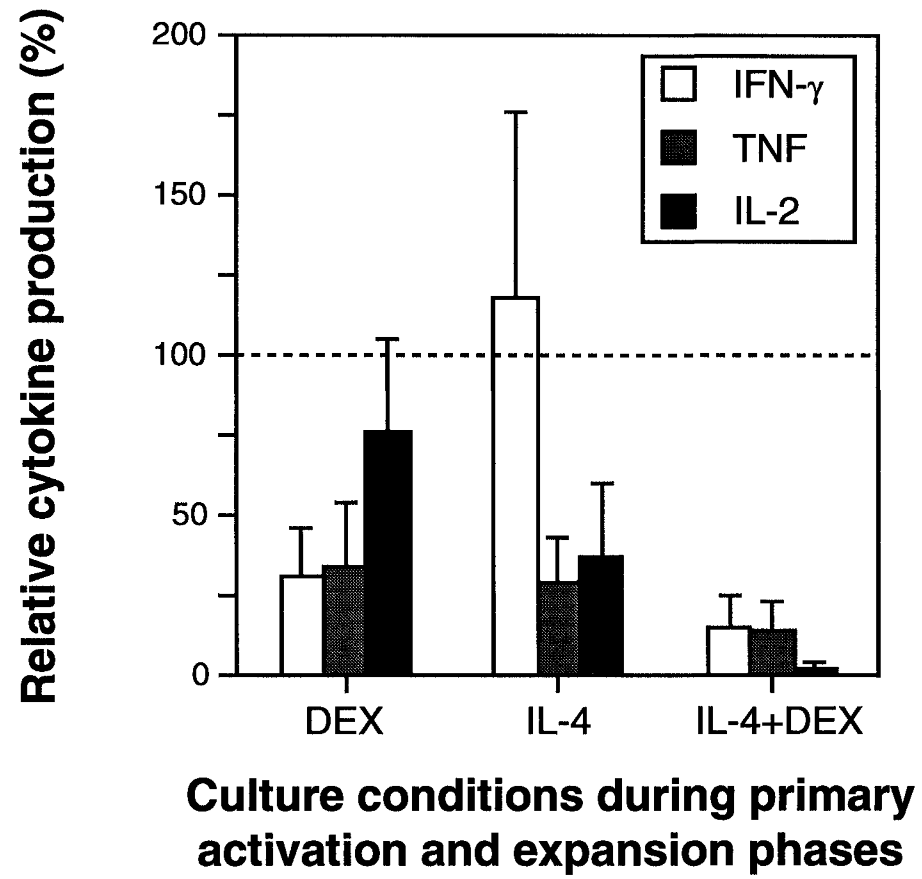

FIGURE 4 The effects of dexamethasone and IL-4 on IFN-γ, IL-2, and TNF synthesis by restimulated CD4+ T cells. Purified rat CD4+ lymphocytes from LNC and thoracic duct were taken through the three-step activation process depicted in Fig. 1. In the last stage, cells were reactivated with Con A in the presence of accessory cells and the levels of IFN-γ, TNF, and IL-2 after 24 hr of the secondary activation were determined as described in Materials and Methods. Results from at least four different experiments are depicted as mean % ± S.D. of the relative cytokine production by the cells in different culture conditions compared to the control incubation (considered 100%; dashed line). Supernatants represented were as follows: DEX (cells activated and expanded in the presence of DEX); IL-4 (cells activated and expanded in the presence of rIL-4); and IL-4+DEX (cultures maintained during the activation and expansion phases with rIL-4 and DEX).

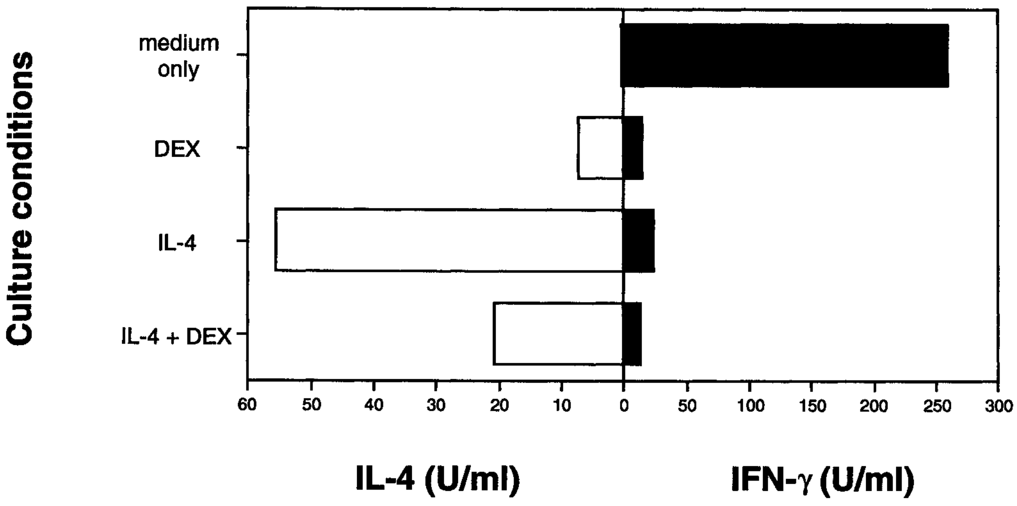

FIGURE 5 The effects of dexamethasone and IL-4 on IFN-γ and IL-4 synthesis by CD4+ T cells stimulated by MLR. Purified DA rat CD4+ lymphocytes from LNC were stimulated with irradiated Lewis splenocytes in the presence of DEX ($10^{-8}$M), IL-4 (500 U/ml), both reagents together, or medium alone. After a 3-day stimulation period, the cells were washed and cultured for 4 days in the presence of IL-2; some cultures were supplemented with different combinations of DEX and IL-4. The cells were recovered and restimulated with irradiated Lewis splenocytes. After 48 hr of the secondary activation, supernatants were removed and the levels of IFN-γ and IL-4 were determined.

IFN-γ production by IL-4 treated cells was observed.

### DISCUSSION

In the present study, we show that the corticosteroid-analogue DEX induces an increase in IL-4 production and a decrease in IFN-γ and TNF synthesis on reactivated CD4+ T cells that had been previously cultured for 1 week in the presence of the hormone. IL-4 and DEX appeared to act synergistically in that together they induced a high level of production of IL-4 and very low levels of IL-2, TNF, and IFN-γ. This is the characteristic pattern of cytokine production by mouse Th2 lines (Mosmann and Coffman, 1989). Two different activation protocols, Con A and MLR, were employed with similar results. The different culture conditions, although showing different pattern of cytokine production, resulted in essentially equally vigorous proliferation in the secondary stimulation (Ramírez et al., 1996). Although not shown, analysis of the production of other Th2 cytokines by reactivated CD4+ T cells have been performed: IL-6 production (mRNA or protein) is not altered by DEX treatment; IL-5 mRNA levels are modestly increased by DEX addition. These results may indicate that the true effect of DEX on the immune response is not to favor the humoral response, but to induce the production of anti-inflammatory cytokines (IL-4, IL-10, and IL-13). This last idea harmonizes well with the physiology of glucocorticoids, controlling the magnitude of the protective responses of the body. Steroids will be released by the adrenal glands during an immune response when the mediators of inflammation reach certain dangerous levels. These hormones have a direct effect on the synthesis of these mediators of inflammation but also may have some indirect and long-lasting effect inducing the synthesis of anti-inflammatory cytokines.

The data support the hypothesis outlined in the Introduction that the autoregulation of EAE is induced by endogenously produced glucocorticoids (Mason, 1991). This hypothesis provides an explanation for the recovery and refractory periods, but a detailed analysis of the cytokines produced during the different phases of EAE, and how endogenous glucocorticoids affect this synthesis, is necessary to demonstrate it. In other systems, a similar effect on cytokine production induced by glucocorticoids has been observed *in vivo* (Daynes and Araneo, 1989; Daynes et al., 1990).

Our demonstration of the modulation of cytokine gene expression by corticosteroids has a number of more general implications. It may be anticipated that endogenous production of these hormones in response to environmental stress may interfere with the immune response required to control infections that normally elicit a protective Th1 type reaction and there are published data to support this suggestion (Sheridan et al., 1991; Moynihan et al., 1994). Such an interaction between the neuroendocrine system and the immune system may, for example, help to explain the prevalence of mycobacterial infections in areas of poverty and malnutrition. Such a possibility has wide implications for the control of such diseases. In addition, our data provide an explanation for how relatively short courses of high-dose steroids used pharmacologically, for example, to control an episode of renal allograft rejection, can apparently have beneficial effects that persist after reduction of the dose of the drug.

IL-4 was included in the experiments as a positive control for the generation of a Th2 cytokine pattern *in vitro* as already has been described (Swain et al., 1990). As expected, we found very potent IL-4 production in the second stimulation and striking inhibition of TNF and IL-2 synthesis in these secondary cultures. However, we did not observe any inhibition of IFN-$\gamma$ production in Con A-activated cells (Figure 4). Increasing the amount of IL-4 in the culture provokes inhibition of IFN-$\gamma$ production in the secondary activation, but this inhibition was never complete (data not shown). In contrast, a strong inhibition of IFN-$\gamma$ production was observed in MLR-activated cells, which indicates differences in the activation signal between the two protocols.

In summary, the present work suggests that corticosteroids not only have a direct anti-inflammatory effect (acute effect), but also promote a long-lasting one, increasing the production of anti-inflammatory interleukins and decreasing the synthesis of proinflammatory cytokines.

### MATERIALS AND METHODS

### Animals

Lewis, DA, and PVG.RT1$^c$ rats were from the specific pathogen-free unit of the MRC Cellular Immunology Unit (Oxford, UK).

### Rat Recombinant Interleukins and Other Reagents

Tissue-culture supernatants containing either rat rIL-2 or rat rIL-4 were obtained from stably transfected CHO cell lines, using the rat IL-2 or rat IL-4 cDNA sequences (McKnight and Classon, 1992). Rat rIFN-$\gamma$ was kindly provided by P. van der Meide (Primate Center TNO, Rijswijk, The Netherlands). Human rIL-2 was from Boehringer Mannheim (Mannheim, Germany). DEX (Sigma, USA) was stored as a stock solution at $10^{-2}$ M in ethanol and diluted into complete medium just before its addition to culture.

### Antibodies

Monoclonal antibodies used were W3/13 (anti-rat CD43) (Williams et al., 1977); MRC-OX6 (anti-rat MHC class II) (McMaster and Williams, 1979); MRC-OX8 (anti-rat CD8) (Brideau et al., 1980); MRC-OX12 (anti-rat $\kappa$ chain) (Hunt and Fowler, 1981), MRC-OX39 (anti-rat IL-2R$\alpha$ chain) (Paterson et al., 1987), and MRC-OX81 (anti-rat IL-4) (Ramírez et al., 1996) were from the MRC Cellular Immunology Unit; DB-1 (mouse anti-rat IFN-$\gamma$ (Van der Meide et al., 1986) was a generous gift of P. Van der Meide (Primate Center TNO, Rijswijk, The Netherlands); polyclonal rabbit anti-mouse IFN-$\gamma$ was kindly provided by J. Tite (Wellcome Research Laboratories, Beckenham, UK); polyclonal rabbit anti-rat TNF was from Innogenetics (Zwijndrecht, Belgium).

### Cells

Lymph node cells (LNC) were obtained from mesenteric and cervical LN. Spleen and LN were removed aseptically from the rats and the cells were isolated by pressing fragments of the tissue through a stainless steel mesh into ice-cold PBS containing 0.2% BSA. Rat thoracic duct lymphocytes (TDL) were obtained by cannulation of the duct (Gowans and Knight, 1964).

### Isolation of Subsets of Lymphocytes

Subpopulations of TDL, LNC, and splenocytes were purified by negative selection using the rosetting technique (Mason et al., 1987). $CD4^+$ T cells were isolated by depletion of B cells, MHC class $II^+$ cells, $CD8^+$ T cells, and cells expressing IL-2R by using the mAbs OX-12, OX-6, OX-8, and OX-39, respectively. T-cell-depleted splenocytes, used as accessory cells in Con A-activated cultures, were prepared by removal of W3/13 positive cells. B cells were purified from TDL by the rosetting technique using the mAb W3/13. After all fractionation procedures cell purities were assessed by labeling pre- and postdepletion samples and analyzing on the FACScan flow cytofluorograph (Becton-Dickinson, USA).

### Cell Culture

To analyze long-lasting effects of GCs on Con A and MLR activation, the protocol used is shown in Figure 1. $CD4^+$ T lymphocytes from LN or thoracic duct were stimulated with Con A in the presence of preirradiated T-cell-depleted splenocytes as accessory cells (Con A activation) or irradiated allogenic splenocytes (MLR activation) in RPMI culture medium supplemented with FCS, $\beta$-ME, and antibiotics. After 3 days in culture (primary activation), the lymphocytes were cultured in the presence of 50 U/ml of rat rIL-2 (expansion phase). Four days later, the cells were centrifuged over a Ficoll-Hypaque density gradient, washed and restimulated with Con A in the presence of irradiated T-cell-depleted splenocytes (secondary Con A activation) or irradiated allogenic splenocytes (secondary MLR activation), and then tested for cytokine production. As indicated in the text and in Figure 1, some cultures were maintained in different combinations of DEX ($10^{-8}$M) and rat rIL-4 (500 U/ml) during the primary activation or expansion phase. In the remaining text of this paper, the three phases of this protocol will be referred to as the primary activation phase, the expansion phase, and the secondary activation phase, respectively. For determination of IL-4, IL-2, TNF, and IFN-$\gamma$ protein production, supernatants were removed at 16 to 24 hr after secondary activation and stored at $-20°C$. RNA extraction (see what follows) was performed after 11 to 24 hr of secondary activation.

### RT-PCR Analysis of Lymphokine mRNA Levels

Total cellular RNA was extracted from $2 \times 10^6$ stimulated $CD4^+$ T cells with RNAzol™ B (Biotecx Laboratories, Houston) according to the manufacturer's instructions, using bacterial ribosomal RNA as a carrier. RNA was reverse-transcribed using oligo-dT as a primer and M-MLV reverse transcriptase (Life Technologies, Paisley, Scotland). The cDNA was serially diluted and IL-4 and $\beta$-actin mRNAs were amplified using the appropriate oligonucleotide primers by PCR for 35 cycles (IL-4 mRNA) or 20 cycles ($\beta$-actin mRNA) in a PTC-100 Programmable Thermal Controller (MJ Research, Watertown, MA). The reaction buffer and the primer sequences for rat IL-4 and $\beta$-actin have been previously described (McKnight et al., 1991). These primers were designed to amplify cDNA fragments of 378 bp for IL-4 and 607 bp for $\beta$-actin. The amplified products were visualized by ethidium bromide staining after agarose-gel electrophoresis. In order to assure that the PCR products were not being assayed on the plateau of the amplification curve, all cDNA samples were amplified at 3 tenfold dilutions. This procedure allowed semiquantitative comparisons to be made of the levels of cDNA present in different samples and ensured that the $\beta$-actin controls were also not run under saturating (plateau) conditions.

### Cytokine Assays

IFN-$\gamma$ was measured by a specific ELISA using the mAb DB-1 (mouse anti-rat IFN-$\gamma$) and a rabbit anti-mouse IFN-$\gamma$ antiserum that cross-reacts with rat IFN-$\gamma$. Rat rIFN-$\gamma$ was used as a standard.

IL-4 protein was measured in the supernatants from activated rat CD4$^+$ T lymphocytes by the increase of MHC class II expression on B cells. To assess the specificity of the assay, the OX-81 mAb was included in some incubations. One unit of IL-4 is defined as the amount of IL-4 that promotes 50% of the maximal class II induction on $5 \times 10^5$ B cells on a volume of 200 $\mu$l.

IL-2 production was measured using the mouse IL-2-dependent cell line CTLL-2 (Gillis et al., 1978). Human rIL-2 was used as a standard. The CTLL-2 cell line responds to rat IL-2 but not to rat rIL-4.

TNF production was measured using TNF-sensitive actinomycin-D-treated murine L929 fibroblasts (Aggarwal et al., 1984). To assess the specificity of the assay, a polyclonal rabbit anti-rat TNF was used and all the activity contained in the supernatants was blocked.

### *Acknowledgements*

I am grateful to Don Mason for discussions during the course of this work and for his critical reading of the manuscript. My thanks are extended to Steve Simmonds and Mike Puklavec for excellent technical assistance and Abdelhadi Saoudi, Ben Seddon, and Phil Stumbles for discussion and assistance. Francisco Ramírez is supported by a Postdoctoral Fellowship from the Multiple Sclerosis Society of Great Britain and Northern Ireland.

### *References*

Aggarwal B.B., Moffat B. and Harkins R.N. (1984). Human lymphotoxin. Production by a lymphoblastoid cell line, purification, and initial characterization. J. Biol. Chem. **259**: 686-691.

Arya S.K., Wong-Staal F. and Gallo R.C. (1984). Dexamethasone-mediated inhibition of human T cell growth factor and $\gamma$-interferon messenger RNA. J. Immunol. **133**: 273-276.

Besedovsky H., Sorkin E., Keller M. and Muller J. (1975). Changes in blood hormone levels during the immune response. Proc. Soc. Exp. Biol. Med. **150**: 466-470.

Besedovsky H.O., del Rey A. and Sorkin E. (1981). Lymphokine-containing supernatants from Con A-activated cells increase corticosterone blood levels. J. Immunol. **126**: 385-387.

Beutler B., Krochin N., Milsark I.W., Luedke C. and Cerami A. (1986). Control of cachectin (Tumor Necrosis Factor) synthesis: Mechanisms of endotoxin resistance. Science **232**: 977-980.

Brideau R.J., Carter P.B., McMaster W.R., Mason D.W. and Williams A.F. (1980). Two subsets of rat T lymphocytes defined with monoclonal antibodies. Eur. J. Immunol. **10**: 609-615.

Byron K.A., Varigos G. and Wootton A. (1992). Hydrocortisone inhibition of human interleukin-4. Immunology **77**: 624-626.

Chikanza I.C., Petrou P., Kingsley G., Chrousos G. and Panayi G.S. (1992). Defective hypothalamic response to immune and inflammatory stimuli in patients with rheumatoid arthritis. Arthrit. Rheum. **35**: 1281-1288.

Daynes R.A. and Araneo B.A. (1989). Contrasting effects of glucocorticoids on the capacity of T cells to produce the growth factors interleukin 2 and interleukin 4. Eur. J. Immunol. **19**: 2319-2325.

Daynes R.A., Araneo B.A., Dowell T.A., Huang K. and Dudley D. (1990). Regulation of murine lymphokine production *in vivo*. III. The lymphoid tissue microenvironment exerts regulatory influences over T helper cell function. J. Exp. Med. **171**: 979-996.

Gillis S., Crabtree G.R. and Smith K.A. (1979). Glucocorticoid-induced inhibition of T cell growth factor production. I. The effect on mitogen-induced lymphocyte proliferation. J. Immunol. **123**: 1624-1631.

Gillis S., Fern M.M., Ou W. and Smith K.A. (1978). T-cell growth factor: Parameters of production and a quantitative microassay for activity. J. Immunol. **120**: 2027-2032.

Gowans J.L. and Knight E.J. (1964). The route of re-circulation of lymphocytes in the rat. Proc. R. Soc. Lond. B. Biol. Sci. **159**: 257-282.

Hunt S.V. and Fowler M.H. (1981). A repopulation assay for B and T lymphocyte stem cells employing radiation chimaeras. Cell Tiss. Kinet. **14**: 445-464.

Kroemer G., Brezinschek H.P., Faessler R., Schauenstein K. and Wick G. (1988). Physiology and pathology of an immunoendocrine feedback loop. Immunol. Today **9**: 163-165.

Levine S., Sowinski R. and Steinetz B. (1980). Effects of experimental allergic encephalomyelitis on thymus and adrenal: Relation to remission and relapse. Proc. Soc. Exp. Biol. Med. **165**: 218-224.

MacPhee I.A.M., Antoni F.A. and Mason D.W. (1989). Spontaneous recovery of rats from experimental allergic encephalomyelitis is dependent on regulation of the immune system by endogenous adrenal corticosteroids. J. Exp. Med. **169**: 431-445.

Mason D.W. (1991). Genetic variation in the stress response: Susceptibility to experimental allergic encephalomyelitis and implications for human inflammatory disease. Immunol. Today **12**: 57-60.

Mason D.W., MacPhee I. and Antoni F. (1990). The role of the neuroendocrine system in determining genetic susceptibility to experimental allergic encephalomyelitis in the rat. Immunology **70**: 1-5.

Mason D.W., Penhale W.J. and Sedgwick J.D. (1987). Preparation of lymphocyte subpopulations. In Lymphocytes: A Practical Approach, Klaus G.G.B., ed. (Oxford: IRL Press), pp. 35-54.

McKnight A.J., Barclay A.N. and Mason D.W. (1991). Molecular cloning of rat interleukin 4 cDNA and analysis of the cytokine repertoire of subsets of CD4$^+$ T cells. Eur. J. Immunol. **21**: 1187-1194.

McKnight A.J. and Classon B.J. (1992). Biochemical and immunological properties of rat recombinant interleukin 2 and interleukin 4. Immunology **75**: 286-292.

McMaster W.R. and Williams A.F. (1979). Identification of Ia glycoproteins in rat thymus and purification from rat spleen. Eur. J. Immunol. **9**: 426-433.

Mosmann T.R. and Coffman R.L. (1989). TH1 and TH2 cells: Different patterns of lymphokine secretion lead to different functional properties. Ann. Rev. Immunol. **7**: 145-173.

Moynihan J.A., Karp J.D., Cohen N. and Cocke R. (1994). Alterations in interleukin-4 and antibody production following pheromone exposure: Role of glucocorticoids. J. Neuroimmunol. **54**: 51-58.

Munck A., Guyre P. and Holbrook N.J. (1984). Physiological functions of glucocorticoids in stress and their relation to pharmacological actions. Endocrin. Rev. **5**: 25-44.

Paterson D.J., Jefferies W.A., Green J.R., Brandon M.R., Corthesy P., Puklavec M. and Williams A.F. (1987). Antigens of activated rat T lymphocytes including a molecule of 50,000 Mr detected only on CD4 positive T blasts. Mol. Immunol. **24**: 1281-1290.

Ramírez F., Fowell D., Puklavec M., Simmonds S. and Mason D. (1996). Glucocorticoids promote a Th2 cytokine response by $CD4^+$ T cells *in vitro*. J. Immunol. **156**: 2406-2412.

Sedgwick J.D., MacPhee I.A.M. and Puklavec M. (1989). Isolation of encephalitogenic $CD4^+$ T cell clones in the rat. Cloning methodology and interferon-$\gamma$ secretion. J. Immunol. Meth. **121**: 185-196.

Sheridan J.F., Feng N., Bonneau R.H., Allen C.M., Huneycutt B.S. and Glaser R. (1991). Restraint stress diferentially affects antiviral cellular and humoral responses in mice. J. Neuroimmunol. **31**: 245-255.

Snyder D.S. and Unanue E.R. (1982). Corticosteroids inhibit murine macrophage Ia expression and Interleukin 1 production. J. Immunol. **129**: 1803-1805.

Sternberg E.M., Young III W.S., Bernardini R., Calogero A.E., Chrousos G.P., Gold P.W. and Wilder R.L. (1989). A central nervous system defect in biosynthesis of corticotropin-releasing hormone is associated with susceptibility to streptococcal cell wall-induced arthritis in Lewis rats. Proc. Natl. Acad. Sci. USA **86**: 4771-4775.

Swain S.L., McKenzie D.T., Weinberg A.D. and Hancock W. (1988). Characterization of T helper 1 and 2 cell subsets in normal mice. Helper T cells responsible for IL-4 and IL-5 production are present as precursors that require priming before they develop into lymphokine-secreting cells. J. Immunol. **141**: 3445-3455.

Swain S.L., Weinberg A.D., English M. and Huston G. (1990). IL-4 directs the development of Th2-like helper effectors. J. Immunol. **145**: 3796-3806.

Van der Meide P., Dubbeld M., Vijverberg K., Kos T. and Schellekens H. (1986). The purification and characterization of rat gamma interferon by use of two monoclonal antibodies. J. Gen. Virol. **67**: 1059-1071.

Waage A., Slupphaug G. and Shalaby R. (1990). Glucocorticoids inhibit the production of IL6 from monocytes, endothelial cells and fibroblasts. Eur. J. Immunol. **20**: 2439-2443.

Williams A.F., Galfre G. and Milstein C. (1977). Analysis of cell surfaces by xenogeneic myeloma-hybrid antibodies: Differentiation antigens of rat lymphocytes. Cell **12**: 663-673.

Wu C.Y., Fargeas C., Nakajima T. and Delespesse G. (1991). Glucocorticoids suppress the production of interleukin-4 by human lymphocytes. Eur. J. Immunol. **21**: 2645-2647.

Zamvil S.S. and Steinman L. (1990). The T lymphocyte in experimental allergic encephalomyelitis. Ann. Rev. Immunol. **8**: 579-621.

